# 

**Published:** 1898-01

**Authors:** 


### CONTENTS:

### SELECTIONS.

Article I.—How to Establish a Dental Practice. By N. S. Hoff, D. D. S. .......... 385
" II.—Use Your Judgment. By Dr. J. Y. Crawford .......... 392
" III.—Evil Effects of Mercury. By R. W. Starr, D. D. S. .......... 393
" IV.—Character in Our Features. By A. O. Hunt, D. D. S. 398
" V.—Report of a Case of Carcinoma of the Buccal Mucous Membrane with Microscopic Exhibit. By Geo. J. Dennis, M. D., D. D. S .......... 399
" VI.—The Etiology of Dental Caries. By D. M. Cathell, D. D. S .......... 402
" VII.—The Bleaching of Discolored Teeth. By J. E. Nyman, D. D. S .......... 406
" VIII.—Successful Method of Filling Fast Decaying Teeth of the Young and Anemic. By C. Bunting Colson, D. D. S .......... 414
" IX.—A Useful Disinfectant .......... 420

### EDITORIAL, ETC.

Dental Examiners of Maryland. Validity of Their Appointment and Their Actions Questioned in a Suit Instituted .......... 422

### OBITUARY.

Dr. William S. McDowell .......... 423

In Memorium.—Dr. Joseph R. Woodley .......... 424

Dr. S. C. Wilkerson .......... 426

The Late Drs. Frank Abbott and L. G. Ingersoll .......... 428

### BIBLIOGRAPHICAL.

The American Text Book of Operative Dentistry .......... 429

### MONTHLY SUMMARY.

Office Drowsiness .......... 431

Deafness .......... 432

